# Investigating MerR’s Selectivity: The Crosstalk Between Cadmium and Copper Under Elevated Stress Conditions

**DOI:** 10.3390/biom14111429

**Published:** 2024-11-09

**Authors:** Anne Soisig Steunou, Anne Durand, Sylviane Liotenberg, Marie-Line Bourbon, Soufian Ouchane

**Affiliations:** Institute for Integrative Biology of the Cell (I2BC), Université Paris-Saclay, CEA, CNRS, 91198 Gif-sur-Yvette, France

**Keywords:** metalloregulator, cadmium/copper, MerR, metal selectivity, metal-binding proteins, CopR, CadR, metal homeostasis

## Abstract

Bacteria respond to metal pollution through sensors that control the uptake and the detoxification machineries. Specificity in metal recognition is therefore a prerequisite for triggering the appropriate response, particularly when facing a mixture of metals. In response to Cu^+^, the purple bacterium *Rubrivivax gelatinosus* induces the efflux Cu^+^-ATPase CopA by the Cu^+^ regulator CopR. However, genetic analyses have suggested the presence of additional regulators. Here, we show that CadR, the Cd^2+^ sensor, is involved in Cd^2+^ and Cu^+^ tolerance and demonstrate that CopR and CadR share common target genes. Interestingly, expression of the Cu^+^ detoxification and efflux (CopI/CopA) system was induced by Cd^2+^ and downregulated in the double mutant *copRcadR^−^*. This double mutant was more sensitive to low Cu^+^ concentration than the single *copR^−^* mutant, and accumulation of coproporphyrin III pointed to a significantly decreased expression of CopA. Furthermore, analyses of Cd^2+^ toxicity in the *cadR^−^* mutant suggested that although CopR is Cu^+^ selective, CopR is involved in Cd^2+^ response since the addition of Cu^+^ alleviates Cd^2+^ toxicity. Based on our current knowledge of metal transport across the inner membrane, Cd^2+^ and Cu^+^ do not share common efflux routes nor do they share common regulators. Nevertheless, the crosstalk between Cd^2+^ and Cu^+^ tolerance systems is demonstrated in the present study. The modulation of Cu^+^ detoxification by a Cd^2+^ regulator in vivo places emphasis on the relaxed selectivity, under elevated metal concentration, in MerR regulators.

## 1. Introduction

Heavy metal pollution is a serious and widespread global environmental problem. Commonly encountered in soil and water, copper (Cu^+^), cadmium (Cd^2+^), lead (Pb^2+^), zinc (Zn^2+^), silver (Ag^+^) and other metals can compromise the survival of microorganisms, unless they have mechanisms allowing them to deal with these metals. Indeed, maintenance of metal homeostasis in cells is critical; it requires a variety of membrane transporters, chaperones, and regulators [1,2]. In most free-living bacteria, the CPx/P_1B_-type ATPase family of heavy metal transporters are the most frequently represented [3,4]. The P_1B_-type ATPase efflux systems remove metal from the cytoplasm to the periplasm where metal is handled by other proteins. However, on the front, detoxification relies first on the effectiveness of sensor-metalloregulators to detect subtle changes in metal content within the cell [2,5]. These metalloregulators modulate gene expression in response to intracellular metal level. In the case of Cu^+^ excess, the CueR (CopR) transcription factor couples Cu^+^ binding to transcriptional activation of the Cu^+^-ATPase pump CopA and of the multi-copper oxidase, if it is present in the genome [6,7]. Furthermore, in a few species, including *Escherichia (E.) coli*, under high Cu^+^ concentration or low Cu^+^ and anaerobiosis, the two-component system CusRS induces expression of the resistance-nodulation-cell division (RND) superfamily CusF/CusCBA efflux system [8]. Similar metalloregulators (MerR family) maintain other monovalent or divalent cation (Ag^+^, Zn^2+^, Cd^2+^, Co^2+^, Hg^2+^, and Pb^2+^) homeostasis, coupling interaction with the metal to the transcriptional induction of genes involved in detoxification and efflux in bacteria [5]. Bacterial genomes contain genes encoding MerR regulators presumably required for the response to elevated levels of different metal ions. These regulators exhibit sequence similarities in their C-terminal metal recognition and binding domain as well as in their N-terminal DNA-binding domain. Yet, these proteins display highly selective recognition of metals in vivo [6,9,10,11]. However, in vitro or under high metal concentrations, metal sensors/regulators can also bind non-cognate metals with lower affinity [10]. The selectivity relies on the metal geometry, its affinity, the ligands in the metal binding site, on the ability of the metalloregulator to bind its target DNA, and on the intracellular metal concentration [10,12]. It was reported that this selectivity relies on the metal concentration in the media and its buffered concentration within the cell [10]. This study concluded that metal sensors are finely tuned to the buffered concentrations of their cognate metal in order to trigger the right response, and when the buffer is saturated under elevated metal concentrations, selectivity is then compromised and metalloregulators can bind non-cognate metals [10].

High concentration of metals, either essential or with unknown biological roles such as Ag^+^, Pb^2+^, or Cd^2+^, are highly toxic and can affect different cellular metabolic pathways. Cu^+^, Ag^+^, and Cd^2+^ exert their toxic effects in *E. coli* partly by disrupting the exposed [4Fe-4S] clusters of dehydratases [13,14]. Cu^+^, to a more severe extent, can compete with iron for the metal binding site in the iron sulfur cluster assembly protein IscA and therefore inhibits the [4Fe-4S] cluster assembly pathway in *E. coli* [15]. Studies in the purple non-sulfur photosynthetic bacterium *Rubrivivax (R.) gelatinosus* and the human pathogen *Neisseria gonorrhoeae* suggest that copper can alter cell growth by affecting heme biosynthesis in the cytoplasm [16,17] or cytochrome *c* assembly in the periplasm [18]. More recently, it was shown that Cu^+^ can also disrupt the activity of the xanthine dehydrogenase required for purine metabolism [19]. All these affected enzymes are part of vital pathways for bacterial fitness and growth.

In *R. gelatinosus*, we identified the Cu^+^-P_1B_-type ATPase CopA, the periplasmic Cu^+^ tolerance protein CopI, and the Cu^+^ sensor regulator CopR [16,18]. Intriguingly, growth of the *copR^−^* mutant was inhibited only at high (500 µM) CuSO_4_ concentration. The induction of CopA expression in response to Cu^+^ in the absence of CopR prompted us to suggest that other putative MerR transcriptional regulators may induce the expression of *copA* under Cu^+^ stress in the absence of CopR [16]. To gain better insights into the mechanism of Cu^+^ tolerance in this bacterium, we aimed to identify the molecular determinants of Cu^+^ resistance in the *copR^−^* mutant. We identified CadR as a response regulator involved in both Cd^2+^ and Cu^+^ tolerance and demonstrated that both CopR and CadR induce *cop* genes expression. CadR is involved in the expression of the Cd^2+^-P_1B_-type ATPase CadA in response to Cd^2+^ and the CopA/CopI system required for copper tolerance in response to Cu^+^ excess. Metal ion selectivity is supposed to avoid cross-recognition and to induce the appropriate response to metal stress. Yet, in the present work, crosstalk between Cu^+^ and Cd^2+^ regulation systems in vivo allows survival under both metal stress conditions. This is highlighted by the fact that cadmium induces Cu^+^ detoxification system and by the finding that copper alleviates the effect of Cd^2+^ in the *cadR^−^* mutant.

## 2. Materials and Methods

### 2.1. Bacterial Strains and Growth

*E. coli* (JM109) was grown at 37 °C in LB medium. *R. gelatinosus* was grown at 30 °C, anaerobically under light (photosynthesis in filled and sealed tubes) on malate medium. Antibiotics were used at the following final concentrations: kanamycin (km), ampicillin, spectinomycin (Sp), streptomycin (Sm), and trimethoprim (Tp) were at 50 µg/mL, and tetracycline was at 2 µg/mL. Bacterial strains and plasmids are listed in Table S1.

Growth curves were monitored at OD_680nm_ with measurements taken every 15 min for 24 h using a Tecan Infinite M200 luminometer (Tecan, Mannerdorf, Switzerland). For photosynthesis conditions, cells were grown anaerobically under light and the OD_680nm_ were measured after 24 h. For the disk diffusion assay, strains were grown to the logarithmic phase (OD_680nm_: 0.8) and 500 μL of cell suspension were added to a cooled soft malate agar (0.5% agar). A sterilized Whatman filter was then added to the solidified agar plates, and 10 µL CuSO_4_ or CdCl_2_ solutions at two different concentrations (100 and 500 µM) were deposited on the filters. The plates were then incubated overnight to assess growth inhibition.

### 2.2. Gene Cloning and Plasmid Constructions for Allele Replacement

Standard methods were performed according to Sambrook et al. [20] unless indicated otherwise. The *cadR* gene was cloned from the genomic DNA of *R. gelatinosus* by polymerase chain reaction using the primers cadR-F and cadR-R (Table S2) into the PCR cloning vectors pGEM-T and pDrive to give *pGcadR* and *pDcadR, respectively.* The *cadR* gene was inactivated by the insertion of Km or Ω (SpSm) cassettes at the StuI site within the *cadR* coding sequence. The resulting recombinant plasmids were designated *pGcadRk* and *pDcadRΩ, respectively.* A 1 kb fragment containing *cadR* was obtained by PCR using the primers cadR_SacI-F and cadR_KpnI-R and cloned in pBBR1MCS-3 at the KpnI-XbaI sites. The resulting plasmid was designated *pB-cadR.*

### 2.3. Gene Transfer and Strain Selection

Transformation of *R. gelatinosus* cells was performed by electroporation. Transformants were selected on malate plates supplemented with the appropriate antibiotic under aerobic conditions. Template genomic DNA was prepared from the ampicillin-sensitive transformants, and confirmation of the antibiotic resistance markers presence at the desired locus was performed using PCR.

### 2.4. Construction of R. gelatinosus cadR^−^ and copRcadR^−^ Strains

pGcadRK and pDcardRΩ plasmids were used to transform the wild type, the *copR*Tp, the *copAH_6_*, the *∆copI-copAH6*, or the *copR^−^-cadAH_6_* to generate the series of mutants described in Table S1. The recombinants were selected aerobically on antibiotics plates and further checked by PCR and on metal containing plates to confirm their metal sensitive phenotype.

### 2.5. Construction of Plasmids for High Level Expression of CadR and CopR in E. coli

His6-tagged constructs were generated using the pET-28b plasmid (Novagen). The genes were amplified by PCR from genomic DNA using the CadR-NdeI and CadR-BamHI primers and CopR-NdeI and CopR-BamHI. NdeI and BamHI sites were introduced at the ATG translation initiation codon and after the TGA stop codon, respectively (Table S2). The NdeI-BamHI PCR fragment was inserted into the NdeI-BamHI site of pET-28b. Recombinant plasmids were isolated and sequenced. The pETcadRH_6_ and pETcopRH_6_ plasmids contain the wild-type sequence of CadR and CopR, respectively.

### 2.6. Overexpression and Purification of His-Tagged Proteins

*E. coli* BL21 (DE3) cells harboring either pETcadRH_6_ or pETcopRH_6_ were grown in LB medium at 30 °C until they reached an OD_600nm_ of 0.6. Then, isopropyl-β-d-thiogalactopyranoside was added to a final concentration of 0.4 mM and incubation was continued for 5 h. Induced cells from 200 mL cultures were resuspended in 50 mL of binding buffer containing 20 mM Tris/HCl (pH 7.9), 5 mM imidazole, and 0.3 M NaCl, and were broken using a French press cell. Unbroken cells and debris were removed by centrifugation at 10,000 rpm for 10 min, and then the soluble fraction was recovered after ultracentrifugation at 45,000 rpm for 1 h, at 4 °C. For purification, the soluble fractions were applied to nickel nitrilotriacetic acid–agarose (Qiagen, Hilden, Germany) affinity columns equilibrated with binding buffer. The column was then washed with the binding buffer and then with the wash buffer (20 mM Tris/HCl (pH 7.9), 0.3 mM NaCl and 20 mM imidazole). The bound proteins were eluted from the column with an elution buffer containing 20 mM Tris/HCl (pH 7.9), 0.3 mM NaCl, and 0.25 M imidazole. The purity of the purified CadR-H_6_ and CopR-H_6_ proteins was checked on a 12% SDS-PAGE stained with Coomassie blue.

### 2.7. Electrophoretic Mobility Shift Assays

One micromole of Cy5-labeled synthetic DNA probes obtained by the annealing of purified oligonucleotides (SIGMA, St. Louis, MO, USA) with 0.5 μg heparin was incubated with purified CadR or CopR in a buffer containing 50 mM Tris HCl at pH 7.5, 50 mM AcK, 20% (*v*/*v*) glycerol, and 1 mM EDTA for 1 h at room temperature. For cadA promoter: a *PcadA* (54 nt) probe was made by annealing the forward primer labeled with Cy5, 5′CCTTTCGTGTCCGACGCTTGACTCTAAACCCGCTACAGGGTCTGCAATGGCGG C3′ and the reverse primer 5′GCCGCCATTGCAGACCCTGTAGCGGGTTTAGAGT CAAGCGTCGGACACGAAA G G 3′. For the copA promoter: a *PcopA* (65 nt) probe was made with the forward primer labeled with Cy5, 5′TTCCGACAGCCCCGCGCTTGACCTTGCCATCGTGGCAAGGTCGACGATGGCTC CGGTCCACAATG 3′ and the reverse primer 5′CATTGTGGACCGGAGCCATCGTCGACCTTGCCACGATGGCAAGGTCAAGCGCGGGGCTGTCGGAA3′. The different DNA/protein complexes were resolved by migration through a 5% (*v*/*v*) 29:1 acrylamide/bisacrylamide gel in 0.5× Tris/borate/EDTA pH 7.6 for 1 h at 150 V at 4 °C. The signals were analyzed using a phospho-imaging system (Molecular Imager^®^ FX; Bio-Rad, Marnes-la-Coquette, France).

### 2.8. Western Blot and Immunodetection

Equal amount of cells (1 OD_680nm_) were disrupted in the loading buffer (10 min at 100 °C), then separated by SDS–PAGE (12% polyacrylamide), and further transferred to a Hybond ECL PVDF membrane, (GE Healthcare, Chicago, IL, USA). Membranes were then probed with the HisProbe-HRP (from Pierce, Appleton, WI, USA) according to the manufacturer instructions, and positive bands were detected using a chemiluminescent HRP substrate according to the method of Haan and Behrmann [21]. Image capture was performed with a ChemiDoc camera system (Biorad) and Image J 1.50d was used for quantification.

## 3. Results

### 3.1. Copper Tolerance Determinants in R. gelatinosus Are Under the Control of at Least Two MerR Regulators

**Figure 1:**
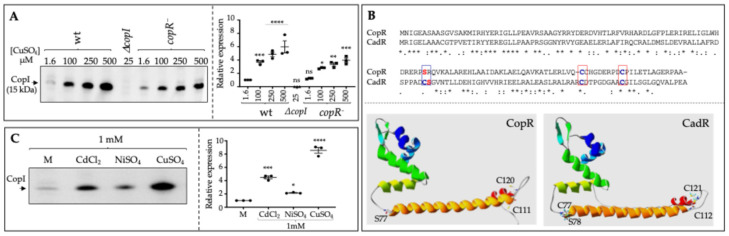
*Metal-dependent induction of CopI protein.* (**A**) Induction of CopI in wild type and *copR^−^* cells in response to increased concentration of CuSO_4_. Total protein extracts from the same number of cells (0.1 OD_680nm_) were separated on 12% SDS-PAGE and CopI was revealed on the Western blot using the HRP-HisProbe (Pierce). (**B**) Sequence alignment and AlphaFold structure model predictions of *R. gelatinosus* CopR and CadR. Cys residues involved in the metal binding are shown in blue in the boxes. The conserved Ser in CopR and in CadR adjacent to Cys79 are shown in red. (**C**) Induction of CopI in wild-type cells in response to 1 mM of CuSO_4_, CdCl_2_, or NiSO_4_. Scatter plots showing the quantitation (ImageJ) of protein amount are shown. Results are the average of 3 different experiments. The quantification is shown as relative to malate (M) medium (1.6 µM CuSO_4_). The results are expressed as the mean ± S.E.M. Significance of variation determined by one-way ANOVA analysis with Dunnet’s Multiple Comparison Test. **** *p* < 0.0001, *** *p* < 0.001, ** *p* < 0.01, * *p* < 0.1, ns non significative.

Copper tolerance in the purple bacterium *R. gelatinosus* involves CopA, which translocates Cu^+^ from the cytoplasm to the periplasm, and CopI, which handles Cu^+^ in the periplasm [16,18]. The induction of CopA involved the MerR Cu^+^ regulator CopR [16]. To confirm the role of CopR in CopI induction as well, the expression of CopI was compared in the wild type and in *copR^−^* strain grown under increasing CuSO_4_ concentration. Thanks to the presence of histidines within the mature CopI sequence, its expression could be detected specifically on Western blots using the (HRP)-conjugated HisProbe [18] and can therefore serve as an accurate reporter protein to assess the metal stress response. As shown in Figure 1A, CopI was gradually induced by CuSO_4_ in the wild type and to a lesser extent in the *copR^−^* mutant. Given that CopA was also expressed in the absence of CopR [16], these data strongly suggest the presence of a second MerR regulator that would control the expression of CopA and CopI. A genome-wide search for metal-responsive regulators revealed the presence of CadR (Zn^2+^/Cd^2+^), ArsR (As^3+^), and NikR (Ni^2+^) sensor regulators. Among these regulators, only CadR showed a sequence (36% identity and 50% homology with CopR (Figure 1B). AlphaFold structure model predictions of CopR and CadR further supported the homologies between the two MerR regulators (https://alphafold.ebi.ac.uk/entry/L0EL98/ https://alphafold.ebi.ac.uk/entry/I0HLS8 (accessed 8 September 2024) [22,23] (Figure 1B).

A closer analysis of the CadR sequence revealed the presence of three conserved cysteines (C79, C114, and C123) involved in Zn^2+^/Cd^2+^ binding [6] in addition to a serine (S80) next to the C79. It is noteworthy that in CopR, Cu^+^ binding requires two cysteines (C112 and C120) and a serine (S77) (Figure 1B). We therefore directly assayed the expression level of the reporter protein CopI in response to 1mM of copper, nickel, or cadmium (Figure 1C). CopI expression was induced in the presence of Ni^2+^ and substantially increased in the presence of Cd^2+^, as previously shown [24].

**Figure 2:**
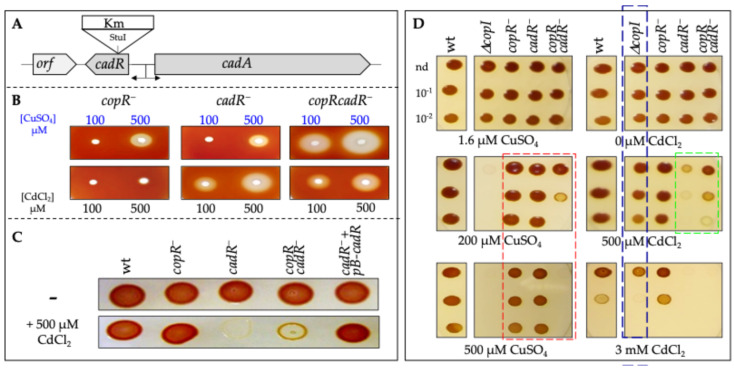
*CadR is involved in both Cu^+^ and Cd^2+^ resistance.* (**A**) Genetic organization of the *cadAR* locus in *R. gelatinosus*. To inactivate *cadR,* the antibiotic resistance km cassette was inserted at the StuI site. (**B**) Cu^+^ and Cd^2+^ toxicity in the *copR^−^, cadR^−^,* and *copRcadR^−^* mutants was assessed on malate agar plates using the disk diffusion assay. The metal-mediated growth inhibition is indicated by clear zones. Plates were incubated under anaerobic photosynthesis for 24 h at 30 °C prior to photography. (**C**) Complementation of the *cadR^−^* strain with the wild type *cadR* gene (*pB-cadR*). Strains were spotted on solid malate medium supplemented or not with CdCl_2_ and incubated under photosynthetic conditions 24 h at 30 °C. The figure highlights also improved growth of the *copRcadR^−^* strain on CdCl_2_ in comparison with the single mutant *cadR^−^*. (**D**) Growth phenotype of the wild type (wt), *ΔcopI*, *copR^−^*, *cadR^−^*, and *copRcadR^−^* mutants in the presence of increasing CuSO_4_ or CdCl_2_ concentrations on solid plates. Plates were incubated under anaerobic photosynthesis for 24 h at 30 °C prior to photography. The red box emphasizes the copper sensitivity in the double mutant *copRcadR^−^*. The blue box emphasizes the cadmium sensitivity of the *ΔcopI* mutant. The green box underlines the cadmium sensitivity of the *cadR^−^* mutant and the unexpected growth recovery in the *copRcadR^−^* double mutant.

To ascertain the putative involvement of CadR in Cu^+^ and/or Cd^2+^ response, we disrupted the *cadR* gene in the wild type and in the *copR^−^* strain (Figure 2A). The requirement for CadR in metal tolerance was first assessed on plates supplemented either with CuSO_4_ or with CdCl_2_ under anaerobic photosynthesis (PS) condition. The disk diffusion assay experiments (Figure 2B) demonstrated that CadR is indeed involved in Cu^+^ tolerance, since the double mutant *copRcadR^−^* was more sensitive to Cu^+^ than the single *cadR^−^* and *copR^−^* mutants. The assay also demonstrated that CadR, but not CopR, is also required for Cd^2+^ tolerance since the single mutant *copR^−^* exhibited no phenotype on CdCl_2_, while the single mutant *cadR^−^* and the double mutant *copRcadR^−^* exhibited the same sensitive phenotype on Cd^2+^. The tolerance towards Cd^2+^ was restored by ectopic expression of the wild-type copy of *cadR* gene (Figure 2C). To support these results, we also spotted cells on agar plate medium supplemented with CuSO_4_ or CdCl_2_ and incubated under anaerobic PS (Figure 2D). *copR*^−^ and *cadR*^−^ strains were able to grow similarly to the wild type in the presence of 500 µM CuSO_4_. In contrast, the double mutant *copRcadR*^−^ strain exhibited decreased tolerance to Cu^+^ as its growth was inhibited by 200 µM CuSO_4_. The *ΔcopI* mutant was used as a control since its growth is completely inhibited at 200 µM CuSO_4_. This confirmed that both CopR and CadR are involved in Cu^+^ tolerance when cells are exposed to 200 µM CuSO_4_. Both the wild type and *copR*^−^ strain grew on plates containing up to 3 mM CdCl_2_. However, the growth of the *cadR*^−^ strain was significantly inhibited on plates with 500 µM CdCl_2_, while the double mutant *copRcadR*^−^ strain exhibited an improved growth on the same plate (Figure 2C,D).

**Figure 3:**
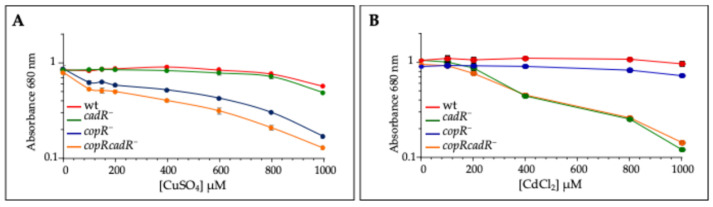
Growth inhibition by increasing CuSO_4_ or CdCl_2_ concentrations in liquid media. wt, *copR^−^*, *cadR^−^* and *copRcadR^−^* mutants were grown over night in the presence of increasing CuSO_4_ (**A**) or CdCl_2_ (**B**) under photosynthesis condition. Growth inhibition curves of the four strains are the average of 3 different experiments. Error bars (some are masked by the point size) represent standard deviation.

Growth inhibition by increased concentration of CuSO_4_ or CdCl_2_ was also measured in liquid medium under anaerobic PS condition (Figure 3). The growth of *copR^−^* and *copRcadR^−^* strains was reduced when exposed to CuSO_4_, while the growth of the wild type and *cadR^−^* strain was unaffected. Yet, the *copRcadR^−^* strain was more sensitive than the single mutant *copR^−^*. In contrast with the plates, the *copR^−^* mutant was more sensitive to CuSO_4_ than the *cadR^−^* mutant. Mutants showing metal sensitivity in the liquid assay but not the agar plates have been reported [25]. In the presence of CdCl_2_, growth of the wild type and *copR^−^* strain was unaffected, while growth of *cadR* and *copRcadR^−^* mutants was reduced (Figure 3B). In agreement with the agar plate data, the double mutant *copRcadR^−^* was more sensitive to CuSO_4_ than the single mutant *copR^−^*, whereas the growth of *cadR* and *copRcadR^−^* strains was comparable in the presence of CdCl_2_ in liquid culture but not on agar plates.

Altogether, these data show that both CopR and CadR are involved in CuSO_4_ tolerance and suggest that CadR, in the absence of CopR, could induce the Cu^+^ tolerance system. Noticeably, the data also show that CadR, but not CopR, is required for CdCl_2_ tolerance and suggest that CopR may interfere with CdCl_2_ tolerance in the absence of CadR, since the double mutant *copRcadR^−^* exhibited enhanced growth on CdCl_2_ on plates.

### 3.2. Unexpectedly, the Cd^2+^ Regulator CadR Controls the Expression Level of the Cu^+^ Tolerance Proteins CopI and CopA

**Figure 4:**
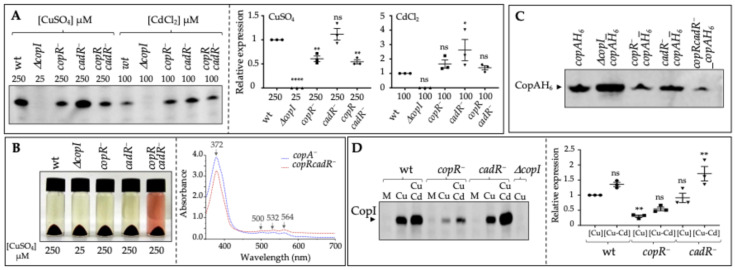
*CadR a Cd^2+^ regulator is involved in CopI and CopA expression in response to excess Cu^+^ in the absence of CopR.* (**A**) Expression of CopI in wild type (wt), *copR^−^*, *cadR^−^,* and *copRcadR^−^* cells in response to CuSO_4_ or CdCl_2_. Cells were grown by photosynthesis and total protein extracts from the same amount of cells (0.1 OD_680nm_) were separated on 12% SDS-PAGE, and proteins were revealed on Western blot using the HRP-HisProbe. Results are the average of 3 different experiments. The quantification is shown as relative to wt stressed cells. The results are expressed as the mean ± S.E.M. Significance of variation determined by one-way ANOVA analysis with Dunnet’s Multiple Comparison Test. (**B**) Effect of CuSO_4_ (250 or 25 µM) on photosynthetic growth in the wt, *ΔcopI*, *copR^−^*, *cadR,^−^* and *copRcadR^−^*. At this concentration, only the *copRcadR^−^* mutant extruded coproporphyrin III in the spent medium. UV-visible absorption spectra of the spent medium from *copA*^−^ and the *copRcadR^−^* cultures confirmed the export of coproporphyrin III into the medium. (**C**) Expression of CopAH_6_ in *R. gelatinosus* wild type (wt), *ΔcopI*, *copR^−^*, *cadR^−^,* and *copRcadR^−^* cells in response to the presence of 250 µM or 25 µM (*ΔcopI*) CuSO_4_ in the growth medium. Results are the average of 3 different experiments and are expressed as the mean ± S.E.M. Significance of variation determined by one-way ANOVA analysis with Dunnet’s Multiple Comparison Test. (**D**) Increased expression of CopI in response to the concomitant presence of 250 µM CuSO_4_ and 200 µM CdCl_2_ in the growth medium. Cells were grown by photosynthesis, total protein extracts from the same amount of cells (0.1 OD_680nm_) were separated on 12% SDS-PAGE, and proteins were revealed on Western blot using the HRP-HisProbe. Results are the average of 3 different experiments. The quantification is shown as relative to malate medium (1.6 µM CuSO_4_). The results are expressed as the mean ± S.E.M. Significance of variation determined by one-way ANOVA analysis with Dunnet’s Multiple Comparison Test. **** *p* < 0.0001, ** *p* < 0.01, * *p* < 0.1, ns non significative.

It is well established that metalloregulatory proteins discriminate between metal ions to trigger the required response according to cell needs. In particular, the MerR regulators are selective and can discriminate between mono and divalent cations [5,6]. Surprisingly, our data suggest that CadR, a divalent cation regulator, can trigger the response to excess Cu^+^, a monovalent cation. To confirm these data, we checked the effect of *cadR* disruption on the expression level of the reporter protein CopI. CopI was induced both in *copR^−^* and *cadR^−^* single mutants in response to CuSO_4_ (Figure 4A). When both *copR* and *cadR* genes were disrupted, the expression of CopI was decreased but not completely abolished. A comparable profile was obtained when cells were challenged with excess CdCl_2_. The fact that CopI was detected in the double mutant in the presence of 250 µM CuSO_4_ or 100 µM CdCl_2_ suggests the involvement of a third metal regulator.

In a previous study, coproporphyrin III was shown to accumulate in the *copA^−^* strain at very low Cu^+^ concentration but only at high concentrations of Cu^+^ in the *copR^−^* strain [16]. Coproporphyrin III accumulates because the 4Fe-4S coproporphyrinogen III oxidase HemN activity is inhibited by the accumulated Cu^+^ in the cytoplasm when the efflux pump CopA is missing or inactive [16,17,26]. To assess the effect of *cadR* inactivation in the *copR^−^* strain on HemN activity, we grew the single and double mutant cells in liquid medium supplemented with 250 µM CuSO_4_ and we analyzed the porphyrin production. As shown in Figure 4B, none of the single mutant *copR^−^* or *cadR^−^* were affected at this Cu^+^ concentration, while in the double mutant, *copRcadR^−^* porphyrin synthesis was very likely to be affected since porphyrin pigment accumulated in the medium. UV-visible absorption spectra (Figure 4B) of the spent medium from the *copRcadR^−^* culture and *copA*^−^ strain used as a reference [16] confirmed that the mutant *copRcadR^−^* mutant accumulated coproporphyrin III. This porphyrin phenotype showed that in the double mutant *copRcadR^−^*, *copA* expression was more affected than in the single mutants, resulting in the accumulation of Cu^+^ in the cytoplasm and the extrusion of coproporphyrin III. These results constitute indirect proof and support the finding that CadR is somehow involved in the expression of CopA in the absence of CopR. To further strengthen these results, strains bearing a His_6_-tag fusion (*copA-H_6_*) gene integrated at the *copA* locus on the chromosome of *R. gelatinosus* [16] were used to assess and compare the amount of CopA in the different strains. As expected, CopA-H_6_ was induced by 250 µM CuSO_4_ in the wild type and 25 µM in the *ΔcopI* strain. In the *cadR^−^* strain, CopA-H_6_ was induced similarly to the wild-type strain, thanks to the presence of CopR (Figure 4C). In the *copR^−^* strain, CopA-H_6_ was still induced but its amount was reduced compared to the wild type, as previously shown in [16]. Inactivation of both regulators in the *copRcadR^−^* double mutant strain resulted in a significant decrease in the amount of CopA-H_6_, thus explaining the porphyrin phenotype of the double mutant challenged with 250 µM CuSO_4_ (Figure 4B,C).

### 3.3. Induction of CopI in Response to the Concomitant Presence of Cu^+^ and Cd^2+^

The induction of CopI in response either to copper or to cadmium prompted us to ask whether these metals and their cognate regulators could synergistically induce CopI expression. In order to check for a possible influence of CdCl_2_ on the expression of CopI in the presence of CuSO_4_, we compared the expression profile of CopI in the wild type and in the different regulator mutants exposed either to 250 µM CuSO_4_ or to 250 µM CuSO_4_ and 200 µM CdCl_2_. The simultaneous presence of Cd^2+^ and Cu^+^ resulted in an increased expression of CopI in the wild type as well as in the single *copR^−^* and *cadR^−^* mutants (Figure 4D). Noticeably, the expression of CopI was increased substantially in the *cadR^−^* background when challenged with both cations, compared to the *copR^−^* strain, suggesting a potential interaction between Cd^2+^ and CopR in the absence of CadR, or the presence of another Cd^2+^ regulator. Together with the genetics and growth inhibition data, these findings demonstrate that the copper tolerance determinants, CopA and CopI, are under the control of at least two MerR regulators: the copper-sensing regulatory protein CopR and the cadmium-sensing regulatory protein CadR, a transcription factor that regulates the cadmium tolerance genes in Gram-negative bacteria. Interestingly, the involvement of *cadR* in Cd^2+^ and Cu^+^ tolerance points to a possible interaction of CadR with both metals, since CadR induces the expression of CopI and CopA in presence of copper.

### 3.4. CadR and CopR Binds In Vitro to the Promoter Region of copA and cadA

**Figure 5:**
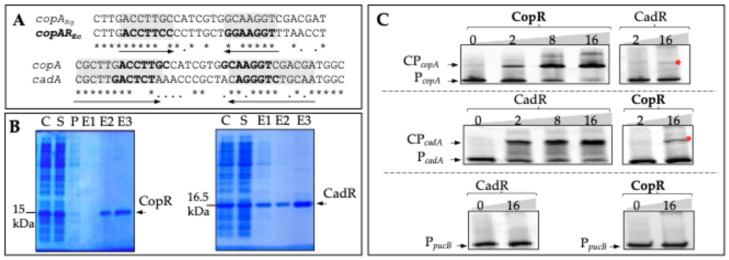
*Electrophoretic gel mobility shift assay with CadR and CopR*. (**A**) Sequence alignments between *copA* promoters from *E. coli* (*copAR_Ec_*) and *R. gelatinosus* (*copA_Rg_*) and between *copA* and *cadA* (*cadA_Rg_*) promoters from *R. gelatinosus.* Similarities between the two regions are indicated with boxes and the putative palindromes are shown with arrows. (**B**) Coomassie Blue of purified CadR and CopR separated on 12% SDS-PAGE. C: cells, S: soluble fraction, P: pass through, E: elution fractions. (**C**) Electrophoretic gel mobility shift assay using fluorescent-labeled PCR fragment from the *copA* (P*_copA_*), *cadA* (P*_cadA_*), and *pucB* (P*_pucB_*) promoter regions with purified CopR or CadR (0 to 16 µg/µL). The resulting DNA/protein complexes CP*_copA_* and CP*_cadA_* were resolved by migration through a 5% 29:1 acrylamide/bisacrylamide gel. The red star shows the DNA/protein complexes between CadR and P*_copA_* or between CopR and P*_cadA_*. P*_pucB_* promoter is used as a negative control.

To demonstrate the interaction between the regulators CopR and CadR, and the promoters of CopA/CopI and CadA, we used the electrophoretic mobility shift assay (EMSA). Alignment of the *R. gelatinosus copA* putative promoter sequence with the *E. coli* CueR binding region (cop-box) showed an inverted repeat sequence within a stretch of 23 bp of *copA* promoter (Figure 5A). A highly similar sequence (cad-box) was also identified in the *cadA* promoter region as revealed by the alignment between the *copA* and *cadA* promoter sequences (Figure 5A). It is hence likely that CadR may recognize and bind *cop* and *cad* boxes, thus activating the transcription of the *copA* and *cadA* genes. For this experiment, we overproduced and purified CopR (15 kDa) and CadR (16.5 kDa) in *E. coli* (Figure 5B). We then performed the EMSA using fluorescently labeled DNA fragments encompassing the promoter region of *copA* (*P_copA_*) or *cadA* (P*_cadA_*). Both regulators (apo-CopR and apo-CadR) bound to the P*_copA_* promoter region, albeit with a much lower affinity between CadR with *P_copA_* (Figure 5C), since the DNA binding target migrates as distinct upper bands in the gel only when apo-CopR or apo-CadR were present in the reaction mixture. CadR and CopR were also able to interact with P*_cadA_* (Figure 5C). In the negative control experiment, neither of the two regulators could interact with the *pucB* promoter. These assays showed that both CopR and CadR can bind in vitro to *copA* promoter, though with different affinities, thus accounting for the induction of the Cu^+^ efflux system in response to CuSO_4_ and CdCl_2_. The data also show that CopR can bind *cadA* promoter. Nevertheless, CopR is not directly involved in cadmium tolerance in response to excess Cd^2+^, as shown above in Figure 2. The sequence similarities between the *cop* and *cad* boxes in the promoter region, together with similarities between CopR and CadR proteins, most likely account for the Cu^+^- and Cd^2+^-induced regulation of the *cop* efflux system.

### 3.5. Copper and Cadmium Cross-Tolerance: Cu^+^ Alleviates the Cd^2+^ Toxicity in cadR^−^ Mutant

**Figure 6:**
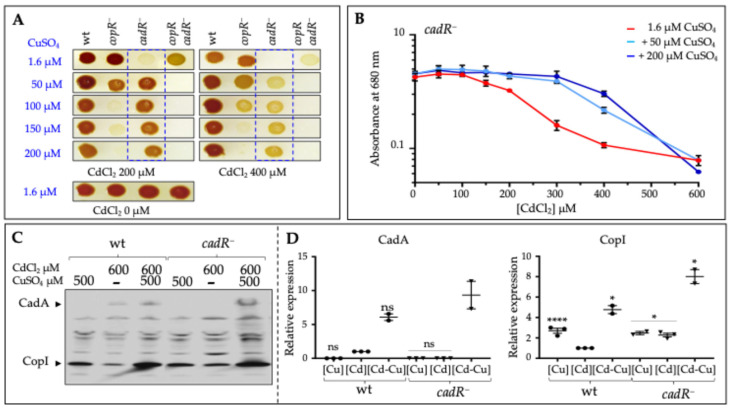
*Copper alleviates the cadmium toxicity in the cadR^−^ mutant*. (**A**) Growth phenotype of the wild type *(wt), copR^−^*, *cadR^−^,* and *copRcadR^−^* mutants in the concomitant presence of CdCl_2_ and CuSO_4_ on solid malate plates. Plates contained either 200 or 400 µM CdCl_2_, with increasing concentrations of CuSO_4_. Plates were incubated under anaerobic photosynthesis for 24 h at 30 °C prior to photography. The blue boxes shed light on the enhanced growth of the *cadR^−^* mutant in the presence of CdCl_2_ when the medium was supplemented with excess CuSO_4_. (**B**) Growth inhibition of *cadR^−^* mutant in CuSO_4_ supplemented medium and in the presence of increasing CdCl_2_ concentrations. Cells were anaerobically grown in liquid by photosynthesis. Results are the average of 3 different experiments. Error bars (some are masked by the point size) represent standard deviation. (**C**) Induction of CadA and CopI expression in the wild type (wt) and *cadR^−^* cells in response to the concomitant presence of CdCl_2_ and CuSO_4_. Cells were grown anaerobically by photosynthesis, and total protein extracts from the same amount of cells (0.1 OD_680nm_) were separated on 12% SDS-PAGE and the proteins were revealed on the Western blot using the HRP-HisProbe. Results are the average of 3 different experiments. (**D**) The quantification is shown as relative to wild-type stressed cells with CdCl_2_. The results are expressed as the mean ± S.E.M. Significance of variation determined by one-way ANOVA analysis with Dunnet’s Multiple Comparison Test. **** *p* < 0.0001, * *p* < 0.1, ns non significative.

The induction of the copper tolerance determinants CopA and CopI by CadR raised the issue of cross-resistance to Cd^2+^ and Cu^+^ in *R. gelatinosus*. Moreover, inactivation of *copR* in the *cadR^−^* background resulted in an increased tolerance to 500 µM CdCl_2_ of the *copRcadR^−^* mutant (Figure 2A,B), suggesting that CopR may contribute to Cd^2+^ sensitivity in the absence of CadR. Given its ability to bind to the *cadA* promoter in vitro, apo-CopR may therefore bind in vivo to this promoter and repress the expression of *cadA* in the absence of its inducer (Cu^+^) and CadR. Thus, in the *cadR^−^* mutant, the addition of CdCl_2_ resulted in growth inhibition when apo-CopR is present. If this hypothesis is valid, then the addition of CuSO_4_ to *cadR^−^* cells when exposed to CdCl_2_ should improve its growth ability. We therefore spotted cells onto agar plates supplemented with CdCl_2_ and increasing concentrations of CuSO_4_ ranging from 1.6 to 200 µM. Excess CuSO_4_ inhibited the growth of *copR^−^* and *copRcadR^−^* strains, as expected (Figure 6A). In the presence of 1.6 µM CuSO_4_, while the *cadR^−^* strain growth was affected by 200 µM CdCl_2_ and completely inhibited by 400 µM CdCl_2_, the double mutant *copRcadR^−^* strain showed improved growth under the same conditions. Interestingly, when grown on plates supplemented with excess (50 to 200 µM) CuSO_4_, the *cadR^−^* strain growth was boosted on plates containing 200 and 400 µM CdCl_2_ (Figure 6A). Under these conditions, the double mutant *copRcadR^−^* strain growth was inhibited by excess CuSO_4_ in agreement with the absence of the two regulators that control the Cu^+^ efflux system. The effect of excess CuSO_4_ on *cadR^−^* strain growth in the presence of Cd^2+^ was also confirmed in liquid cultures by analyzing the growth inhibition in the presence of both cations. The addition of 50 µM CuSO_4_ resulted in an improved growth of *cadR^−^* in the presence of increasing CdCl_2_ concentration between 50 and 400 µM (Figure 6B). Similarly, the addition of 200 µM CuSO_4_ improved growth of *cadR^−^* in CdCl_2_ up to 400 µM. Beyond 600 µM of CdCl_2_, the addition of CuSO_4_ was toxic and *cadR^−^* strain growth was inhibited. These results showed that the low concentration of CuSO_4_ alleviates CdCl_2_ toxicity in the *cadR^−^* mutant, thus revealing a crosstalk between Cu^+^ and Cd^2+^ regulatory systems in this bacterium. We have previously reported this interesting crosstalk between Cd^2+^ and Cu^+^ in *R. gelatinosus* [24] in the wild type strain; here we show that it relies on the regulatory proteins and very likely their ability to induce the efflux systems.

### 3.6. The Enhanced Growth in the Presence of CdCl_2_ and CuSO_4_ Is Associated with an Increased CadA Expression

The growth recovery in the combined presence of CdCl_2_ and CuSO_4_ in the *cadR^−^* strain could be interpreted as an induction of the copper tolerance system CopA/CopI, which would detoxify Cd^2+^ from the cells, or other proteins that detoxify Cd. It could also be the consequence of the repression removal of *cadA* expression by CopR. CopI and CopA are indeed somehow involved in Cd^2+^ tolerance, but only in the *ΔcadA^−^* strain [24]. Conversely, induction of CadA by the addition of CuSO_4_, in the absence of CadR, may account for the observed growth recovery of the *cadR^−^* strain. To confirm this, we checked by Western blot the expression level of CadA and CopI in the wild-type and *cadR^−^* strains grown in the presence of a high concentration of CdCl_2_ supplemented or not with 500 µM CuSO_4_. Like CopI, because of the presence of several histidine in its C-terminal sequence, CadA can also be directly detected with the HisProbe [24]. As shown in Figure 6C, in the wild type cells, a low amount of CadA is detected in the presence of 600 µM of CdCl_2_. The addition of 500 µM CuSO_4_ resulted in an increase in CadA expression and CopI level. In contrast, in the *cadR^−^* mutant, CadA was not detected in the presence of 600 µM of CdCl_2_, yet CopI was induced in these cells (Figure 6C). Interestingly, addition of 500 µM of CuSO_4_ resulted in a substantial increase in the expression of CadA and CopI in the *cadR^−^* mutant in the presence of 600 µM CdCl_2_ (Figure 6C). In the control experiment in which cells were exposed either to 600 µM of CdCl_2_ or to 500 µM of CuSO_4_, CopI was expressed but not CadA. These findings are consistent with the enhanced growth of the *cadR^−^* strain in presence of CdCl_2_ and CuSO_4_ as a consequence of CadA induction.

### 3.7. Cd^2+^ and Cu^+^ Tolerance in R. gelatinosus Involves Two Distinct ATPases but Share CadR, a Common Sentinel to Deal with Excess Cd^2+^ and Cu^+^

**Figure 7:**
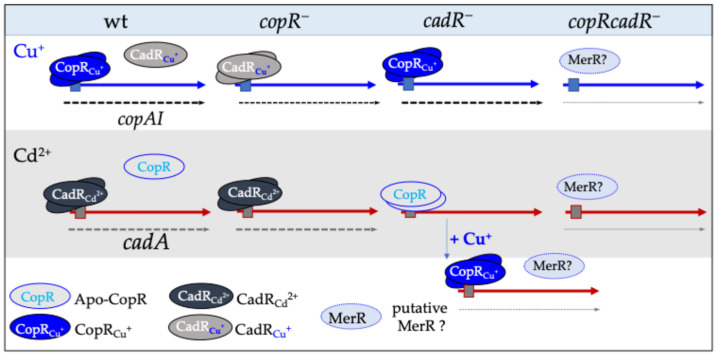
*Schematic putative model of crosstalk between the copper and cadmium regulons in R. gelatinosus*. Both CopR and CadR can induce the expression of *copAI* in the wild type. CadR is required for the expression of *cadA*, and apo-CopR is proposed to repress *cadA* expression. Copper addition leads to the expression of *cadA,* thus revealing the crosstalk between the two metal regulatory systems. Expression of *copAI* in the double mutant points to a third putative regulator. For simplification, detoxification coding genes are represented by blue (*copAI*) or red (*cadA*) arrows, protein names are given, the dashed lines represent transcriptional activation, and the thickness reflects the relative expression. Direct interaction between the regulators and metals was not demonstrated at this stage.

The findings reported above demonstrate a link between copper and cadmium control of the copper tolerance detoxification system. This raised the question of whether the system is also involved in Cd^2+^ detoxification. At this stage, only CopI seems to be involved in this mechanism [24]. Nevertheless, additional investigations are required to support the genetics data. On the contrary, the induction of CopA and CopI by Cd^2+^ is clearly demonstrated and very likely arises from the similarities on one hand between the promoter sequences of *copA* and *cadA*, and on the other hand between CopR and CadR proteins. Our results support a putative model (Figure 7) indicating that in the wild type, in presence of copper, the expression of *copA* and *copI* are presumably induced by CopR but also by CadR, as evidenced by the ability of CadR to induce CopA and CopI expression in response to Cu^+^ or Cd^2+^ in the absence of *copR* (*copR^−^*). In the absence of CadR (*cadR^−^*), the expressions of *copA* and *copI* are under the control of CopR, while the expression of *cadA* is somehow down-regulated by CopR, as evidenced by the ability of the double mutant *copRcadR^−^* to achieve growth on Cd^2+^ following the addition of Cu^+^, which alleviates the Cd^2+^ inhibition growth phenotype. This could be the result of the Cu^+^ binding by CopR or by a yet unidentified additional transcriptional factor (Figure 7). The presence of an additional factor that contributes to *copA* and *copI* gene expression is suggested by the ability of the *copRcadR^−^* double mutant to grow on copper and to induce *copA* and *copI* expression. This speculative and tentative model requires transcriptomic analyses to confirm the transcriptional activation of the target genes. Nonetheless, quantitative differential proteomics (to be published) analysis of cells grown either with excess Cu or with Cd confirmed the induction of CopA and CopI by CdCl_2_, and the Cd^2+^ efflux pump CadA by CuSO_4_.

## 4. Discussion

Metal homeostasis relies on a robust arsenal of sensors/regulators able to selectively recognize and interact with a metal to induce or repress the expression of the detoxification systems [1,2,3,9]. Structural elements involved in the metal selectivity of metal-responsive transcriptional regulators in bacteria have been studied to understand the remarkable selective feature very often encountered in the majority of regulators [6,9,10,11,27,28]. There are however a few exceptions in the literature to this strong selectivity, where a given regulator could respond to several metals with different valences [9,10,29,30,31,32,33,34]. This relaxed selectivity is also evident in *R. gelatinosus* for two MerR regulators, CadR involved in the activation of the Cd^2+^ detoxification system and CopR involved in the activation of the Cu^+^ homeostasis system. Indeed, in this study, we have shown that CadR is involved in the expression of CopA and CopI in the presence of copper, and that CopR is somehow involved in CadA expression and Cd^2+^ tolerance. How can a Cd^2+^ sensor–regulator respond to Cu^+^ in this bacterium and other species? Comparison of the CueR (CopR) and ZntR (CadR) crystal structures revealed some determinants of metal-ion selectivity [6]. It was proposed that Ser77 can establish hydrogen bond interactions with several main chain atoms within the Cu-binding loop of CueR (CopR). By these means, Ser77 could be involved in the conformation of the Cu-binding site [6]. Moreover, it was suggested that this Ser77 is important in discriminating between mono and divalent cations. A serine is found at this position in all MerR homologs responsive to monovalent ions, whereas a cysteine is present in MerR, which responds to divalent ions [6]. In *R. gelatinosus*, in the CadR, the presence of serine Ser80 close to cysteine Cys79 could be at the origin of structural readjustments within the Cd^2+^ binding loop in the presence of Cu^+^, hence stabilizing Cu^+^ binding to CadR. Furthermore, in *Salmonella typhimurium*, it was shown that mutation of Ser77 to Cys77 within the Cu^+^ CueR regulator gave rise to a regulator (CueRS77C) that binds and responds to both Cu^+^ and divalent cations including Cd^2+^ or Zn^2+^ in the *zntA* mutant accumulating Cd^2+^ or Zn^2+^ [30]. These data show that selectivity in CopR can indeed be affected by this Ser in the vicinity of the metal binding site. Other exceptions to this strong selectivity are present in other bacteria. In *Bacillus (B.) subtilis*, the expression of the Cu^+^-efflux system CopZA is under the control of CueR. *copZA* expression is also induced in response to elevated concentrations of Cd^2+^ ions [32,33]. Similarly, in *B. subtilis,* the copper sensor repressor CsoR binds Cu^+^ with very high affinity but also divalent ions such as Ni^2+^, Zn^2+^, and Co^2+^ [9]. In the cyanobacterium *Osciliatoria brevis*, the BxmR regulator, part of the ArsR family metal sensor proteins, regulates the expression of the P-type ATPase Bxa1 and the metallothionein BmtA in response to Cu^+^, Ag^+^, Zn^2+^, and Cd^2+^ [31,34]. Interestingly and quite unexpectedly, in *Pseudomonas (P.) aeruginosa,* Cu^+^ stress induced resistance to Zn^2+^. Molecular analyses showed that CopR, a Cu^+^ two-component system response regulator, induced the expression of the CzcRS two-component system that controls the expression of the CzcCBA efflux pump required for Zn^2+^, Cd^2+^, and Co^2+^ detoxification [29]. In most cases, the physiological significance of the crosstalk between metal regulatory systems remains unclear. Indeed, only the P-type ATPase Bxa1 was shown to be induced and to confer resistance to both monovalent and divalent cations [34]. On the contrary, in *B. subtilis* and *R. gelatinosus*, Cd^2+^ induces *copA* expression [24], yet the efflux pump is specific to monovalent ions [32,35,36]. Likewise, in *P. aeruginosa*, although CzcCBA is induced by Cu^+^, it remains specific for Zn^2+^, Cd^2+^, and Co^2+^ efflux [29]. Altogether, these reports show that although very selective, metal regulators can bind non-cognate metal ions and induce nonspecific responses under elevated metal concentrations. The work by Osman and coworkers also showed a relaxed selectivity of metal regulators and pointed out the importance of the intracellular buffer in this mis-metalation and triggered response. Their work revealed that metal selectivity is limited to a finely controlled range of buffered metal concentrations [10]. *R. gelatinosus* CadR is another exception to the high selectivity of the MerR metal-responsive transcriptional regulator family in bacteria under copper excess. Structural insights into CadR protein in the presence of Cu^+^ or Cd^2+^ would be of great significance and will provide structural details as to how originally very selective metal-responsive transcriptional regulators can evolve to give rise to less selective proteins. It is tempting to speculate that this relaxed selectivity can provide bacteria with a selective advantage. This may be the case in the environment, particularly in polluted areas, in which microorganisms are exposed to a mixture of metals. Growing new evidence is now indicating potential cross-talk between Cu^+^, Cd^2+^, Zn^2+^, Mn^2+^, and Fe^2+^ homeostasis systems in bacteria [24,37,38,39]. It is hence important to study and understand the biological effects of such mixtures and the mechanisms of toxicity and response. Our group and others have shown that Fe^2+^ partially alleviates Cu^+^ or Zn^2+^ toxicity, respectively, in *R. gelatinosus* and *E. coli* [39,40]. More recently, Hong et al. showed that supplementation with Zn^2+^ or Mn^2+^ partially alleviates Cu^+^ stress in *Streptococcus pyogenes* [38]. Here, we show crosstalk between Cu^+^ and Cd^2+^ homeostasis systems, demonstrating that metal mixtures could, to some extent, represent a selective advantage in the environment since supplementation with Cu^+^ partially decreased Cd^2+^ toxicity.

Significance and Conclusion: Cu^+^ and Cd^2+^ have been widely dispersed into the environment through anthropogenic activities. To deal with these metals in their environment, bacteria discriminate between ions and trigger the required response according to cell needs, thanks to the high selectivity of metal-responsive transcriptional regulators. However, this selectivity can be undermined in the event of excess or mixtures of metal ions. Our findings provide insights into the crosstalk between the Cu^+^ and Cd^2+^ detoxification systems in vivo, and the adaptation of *Rubrivivax gelatinosus* to Cu^+^ and Cd^2+^ through MerR regulators with relaxed selectivity. How microorganisms cope with excesses and mixtures of metals in their environment is important to our basic understanding of microbial ecology, but also to promote efficient and sustainable bioremediation strategies of metal contaminated soils.

## Data Availability

Data are contained in the article and Supplementary Materials.

## Supplementary Materials

The following supporting information Table S1: Bacterial strains and plasmids, Table S2: Primers can be downloaded at https://www.mdpi.com/article/10.3390/biom14111429/s1 [41,42,43,44].
