# Supplementary material for: Investigating MerR’s Selectivity: The Crosstalk Between Cadmium and Copper Under Elevated Stress Conditions
Source: Biomolecules. 2024 Nov 9;14(11):1429. doi: 10.3390/biom14111429 (PMC11591864; doi:10.3390/biom14111429)
Supplement: Supplementary file 1 [file biomolecules-14-01429-s001.zip › biomolecules-3236035-supplementary.pdf]

Table S1: Bacterial strains and plasmids

	**Relevant characteristics**	**Source**
**Strains**		
E. coli		
JM109	*el4$^-$(McrA$^-$), recA1, endA1 gyrA69, thi-1, hsdR17 (rk- mk+) supE44, reA1, Δ(lac-proAB) [F'traD36, proAB, lacIZΔM15*	Stratagene
BL21	*B F$^-$ ompT gal dcm lon hsdS$_B$(r$_B^-$m$_B^-$) [malB$^+$]$_{K-12}$(λ^S)*	Novagen
R. gelatinosus		
Strain S1	Wild type (wt)	[41]
cadR$^-$	*cadR* inactivated strain (*cadR::K*)	This work
*cadR*Ω	*cadR* inactivated strain (*cadR::*Ω)	This work
ΔcopI	*copI* inactivated strain (*copI::Km*)	[18]
copR$^-$	*copR* inactivated strain (*copR::Tp*)	[16]
copR$^-$cadR$^-$	*copR* (*copR::Tp*) and *cadR* (*cadR::Km*) inactivated strain	This work
*copR$^-$cadR*Ω	*copR* (*copR::Tp*) and *cadR* (*cadR::* Ω) inactivated strain	This work
copAH$_6$	Strain bearing the His$_6$ tagged *copA* gene	[16]
ΔcopI-copAH$_6$	*ΔcopI::Tp* bearing the His$_6$ tagged *copA* gene	[18]
copR$^-$-copAH$_6$	*copR::Tp* bearing the His$_6$ tagged *copA* gene	[16]
*cadR*Ω*-copAH$_6$*	*cadR::*Ω bearing the His$_6$ tagged *copA* gene	This work
*copR$^-$cadR*Ω*-copAH$_6$*	*copR::Tp_cadR::*Ω strain bearing the His$_6$ tagged *copA* gene	This work
**Plasmids**		
pGEM-T	Cloning vector (Ap^r)	Promega
pDrive	Cloning vector (Ap^r Km^r)	Qiagen
pUC4K	Plasmid bearing the Km cartridge (Ap^r Km^r)	Pharmacia
pDW9	Plasmid bearing the cartridge (Sp-Sm^r)	[42]
p34S-Tp	Plasmid bearing the Tp cartridge (Ap^r Tp^r)	[43]

pGcadR	pGEM-T + 1kb PCR fragment containing *cadR*	This work
pGcadRK	Km cartridge cloned into StuI site in pGcadR	This work
pDcardR	pDrive + 1kb PCR fragment containing *cadR*	This work
pDcadRΩ	Ω cartridge cloned into stuI site in pDcadR	This work
pETcadRH$_6$	*cadR* cloned in the pET28b plasmid at NdeI-BamHI	This work
pETcopRH$_6$	*copR* cloned in the pET28b plasmid at NdeI-BamHI	This work
pCopAH$_6$	*copA* cloned in the pET28b plasmid at the EcoRI-XhoI	[16]
pBBR1MCS-3	(mob$^+$, Tc^r) expression vector	[44]
pB-cadR	*cadR* gene cloned in pBBR1MCS-3 SacI-KpnI sites	This work

Ap^r, ampicillin resistant, Sp-Sm^r streptomycin, spectinomycin resistant, Km^r, kanamycin resistant, Tp^r, trimethoprim resistant, Tc^r, tetracyclin resistant.

Table S2: Primers used in this work

Primer	Sequence 5′to 3′
cadRF	ACGACGACGAGGAGTTCGTCGA
cadRR	GATCTGCCCTTCCTCAGACGGCGCAG
cadR_SacI_F	GTGATCGTGCGAGCTCGTTCGTGCGCGGG
cadR_KpnI-R	GCACGAGTCGGTACCTGCACTACGGCCAG
cadR-NdeI	CGGACACGAAAGGCATATGGCGATGCGCATC
cadR-BamHI	CTGAGCCCGCGCGGATCCTTCAGCGCCGCC
copR-NdeI	GCCGGAGAGTCGCATATGAACATCGGCG
copR-BamHI	GCGGCAAGCGTAGGATCCGCGCCGGCTCAG

Whole Western blots from **Figure 1** showing all bands.

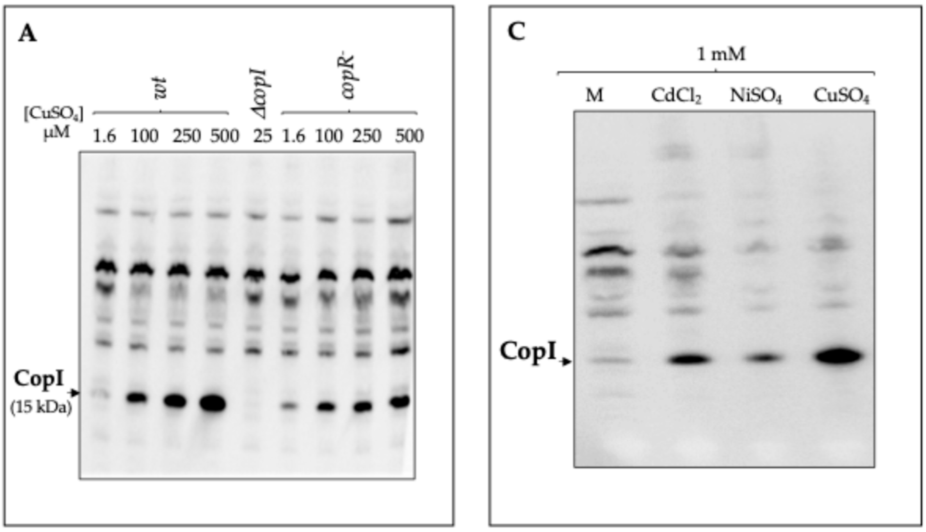

Fig. 1: Expression of CopI in response to metal ions

Whole Western blots from **Figure 4** showing all bands.

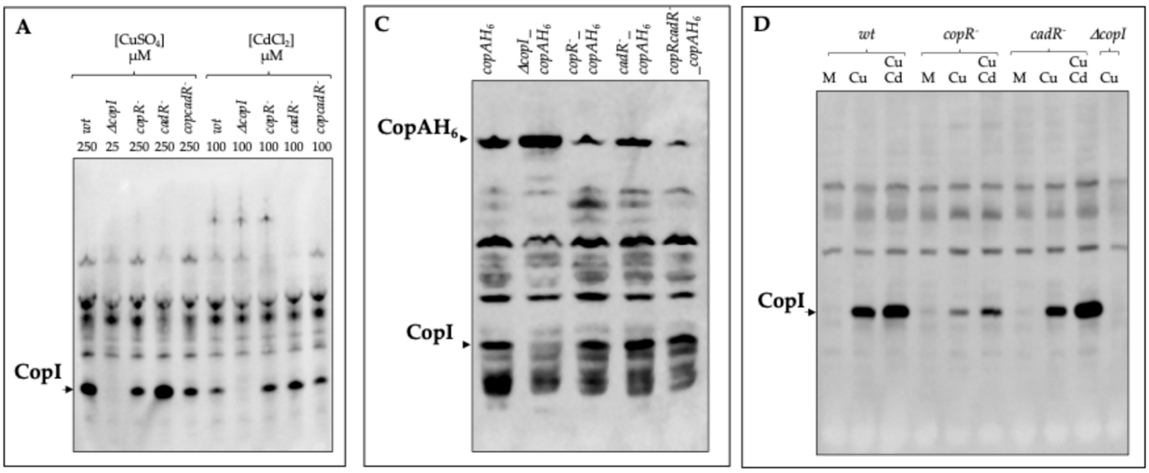

Fig. 4: Expression of CopI and $CopAH_6$ in response to Cu and Cd ions in different genetic backgrounds

Whole Western blots from **Figure 6** showing all bands.

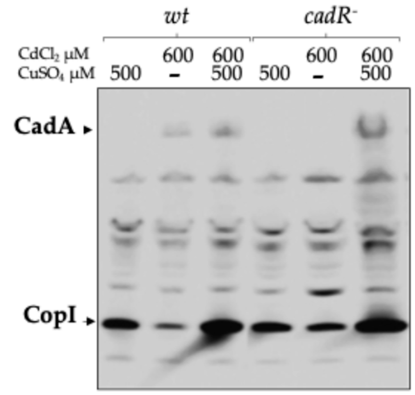

Fig. 6: Expression of CopI and CadA in response to Cu and Cd ions in wt and cadR⁻ mutant.

Whole EMS assay from **Figure 5** showing all bands.

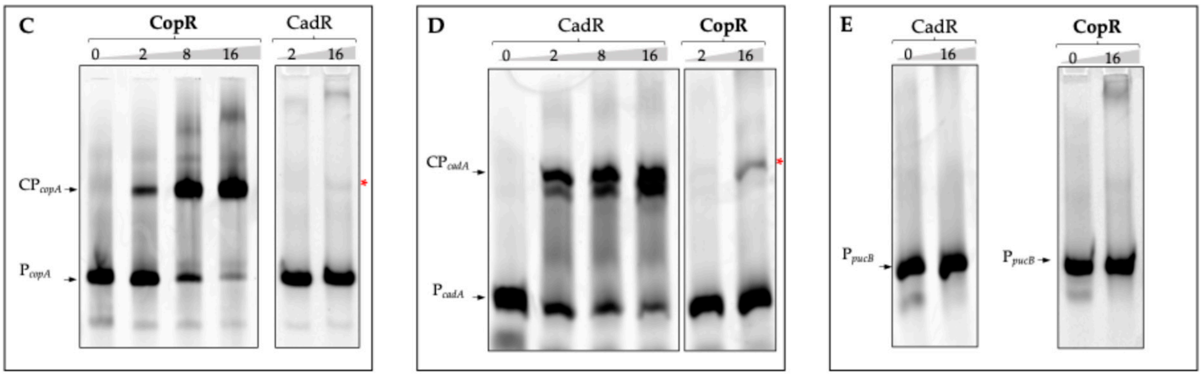

Fig. 5: Electrophoretic gel mobility shift assay with CadR and CopR

16 Azzouzi, A.; Steunou, A.S.; Durand, A.; Khalfaoui-Hassani, B.; Bourbon, M.L.; Astier, C.; Bollivar, D.W.; Ouchane, S. Coproporphyrin III excretion identifies the anaerobic coproporphyrinogen III oxidase HemN as a copper target in the Cu^{+}-ATPase mutant *copA⁻* of *Rubrivivax gelatinosus*. *Mol. Microbiol.* **2013**, *88*, 339–351.
18 Durand, A.; Azzouzi, A.; Bourbon, M.L.; Steunou, A.S.; Liotenberg, S.; Maeshima, A.; Astier, C.; Argentini, M.; Saito, S.; Ouchane, S. c-type cytochrome assembly is a key target of copper toxicity within the bacterial periplasm. *mBio* **2015**, *6*, e01007–e01015.

41 Uffen, R.L. Anaerobic growth of a Rhodopseudomonas species in the dark with carbon monoxide as sole carbon and energy substrate. *Proc. Natl. Acad. Sci. USA* **1976**, *73*, 3298–3302. [CrossRef]
42. Prentki, P.; Krisch, H.M. In vitro insertional mutagenesis with a selectable DNA fragment. *Gene* **1984**, *29*, 303–313.
43. Dennis, J.J.; Zylstra, G.J. Plasposons: Modular self-cloning minitransposon derivatives for rapid genetic analysis of gram-negative bacterial genomes. *Appl. Environ. Microbiol.* **1998**, *64*, 2710–2715.
44. Kovach, M.E.; Phillips, R.W.; Elzer, P.H.; Roop, R.M., 2nd; Peterson, K.M. pBBR1MCS: A broad-host-range cloning vector. *Biotechniques* **1994**, *16*, 800–802.
